# Excited States of Xanthophylls Revisited: Toward the Simulation of Biologically Relevant Systems

**DOI:** 10.1021/acs.jpclett.1c01929

**Published:** 2021-07-12

**Authors:** Mattia Bondanza, Denis Jacquemin, Benedetta Mennucci

**Affiliations:** † Dipartimento di Chimica e Chimica Industriale, University of Pisa, via G. Moruzzi 13, 56124 Pisa, Italy; ‡ Université de Nantes, CNRS, CEISAM UMR 6230, F-44000 Nantes, France

## Abstract

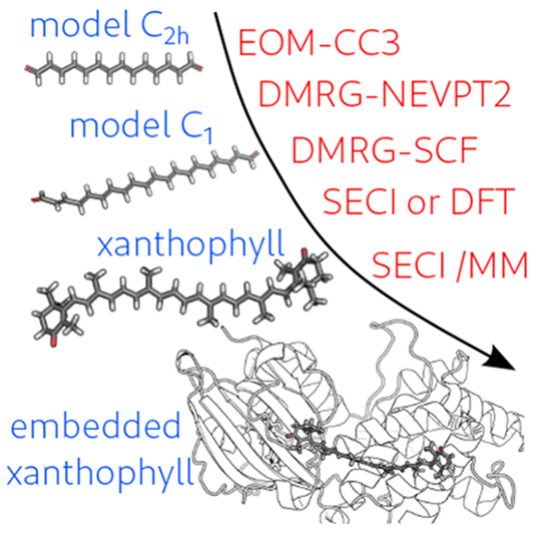

Xanthophylls are a class of oxygen-containing carotenoids, which play a fundamental role in light-harvesting pigment–protein complexes and in many photoresponsive proteins. The complexity of the manifold of the electronic states and the large sensitivity to the environment still prevent a clear and coherent interpretation of their photophysics and photochemistry. In this Letter, we compare cutting-edge *ab initio* methods (CC3 and DMRG/NEVPT2) with time-dependent DFT and semiempirical CI (SECI) on model keto-carotenoids and show that SECI represents the right compromise between accuracy and computational cost to be applied to real xanthophylls in their biological environment. As an example, we investigate canthaxanthin in the orange carotenoid protein and show that the conical intersections between excited states and excited–ground states are mostly determined by the effective bond length alternation coordinate, which is significantly tuned by the protein through geometrical constraints and electrostatic effects.

Carotenoids are ubiquitous compounds in living organisms where they contribute to the functions of different pigment–protein complexes.^1−3^ This large diffusion and the multiple roles they play stem from their unique characteristics of being both “extensible” and “flexible” molecules, with electronic and optical signatures which are strongly interlinked with their length and conformation. These characteristics render the properties of carotenoids highly tunable depending on the specific embedding environment.

Many of the biological functions involving carotenoids exploit the specific photophysics brought by their low-lying π → π* excited-state manifold. Illustratively, in an idealized perfectly symmetric *C*_2*h*_ polyene, the two lowest excited states that are typically investigated are the optically dark *A*_*g*_ and bright *B*_*u*_. However, another *B*_*u*_ state is present among the low-lying π → π* states but with a dark character. To distinguish between the two, the notation *B*_*u*_^+^ (bright) and *B*_*u*_^–^ (dark) derived from particle–hole symmetry^4^ is often used. Moreover, in carotenoids containing carbonyl groups, additional states of n → π* character are present. In *C*_2*h*_ symmetric cases, the two lowest n → π* states belong to the *A*_*u*_ and *B*_*g*_ representations.

The strong absorption of carotenoids arises from a transition from the ground to the *B*_*u*_^+^ state, which, by internal conversion, rapidly decays (in less than 200 fs) to *A*_*g*_ state. The latter next decays to the ground state by internal conversion in several picoseconds and fluorescence consequently occurs with an extremely low quantum yield. The presence of another “dark S*” state in the vicinity of *A*_*g*_ and *B*_*u*_^+^ has been also suggested.^5,6^ However, the nature and energetic position of this S* state remains unclear, and there are debates regarding its nature: is it a real, separate electronic excited state or, alternatively, a twisted *A*_*g*_ state or even a vibrationally hot ground state?^7,8^ Finally, in xanthophylls, a further complexity has been suggested, namely, that the excited-state relaxation may proceed through the coupling with an intramolecular charge transfer (ICT) state depending on solvent polarity.^9^

From this brief summary, it is clear that, despite the large interest in the photophysics of carotenoids, a clear and established theoretical characterization of their excited-state manifold is still missing. The main reason is that applying accurate *ab initio* QM methods to the study of carotenoids remains challenging. Indeed, the delicate coupling between structural and electronic degrees of freedom, combined with the very different nature of the key excited states, renders the complete characterization of these systems feasible for “short” carotenoids only,^10,11^ which are not those of real biological interest. We note, however, that a very recent paper by Khokhlov *et al.*(12) has shown that a balanced picture of the low-lying excited states of large polyenes can be achieved using driven similarity renormalization group-multi reference perturbation theory (DSRG-MRPT2).^13,14^ In any case, the realistic simulation of the photophysics of carotenoids within their biological environment requires the inclusion of dynamics, thus making the application of highly accurate *ab initio* methods even more difficult if not impossible.

The most obvious computational choice to explore excited states of medium-large molecules is time-dependent density functional theory (TD-DFT), which is the gold standard in terms of cost-effectiveness. TD-DFT, however, has the strong limit of being a single-reference method, and as such, it cannot properly describe states with a significant multireference character and/or states having a significant double-excitation character. This is indeed the case for the *A*_*g*_ state in which the doubly excited configurations play a significant role, meaning that conventional TD-DFT overshoots its transition energy, in contrast to the *B*_*u*_^+^ state that is reasonably well captured by TD-DFT. A possible strategy to overcome this limit is to combine DFT with a multireference configuration interaction (MRCI) ansatz; the resulting DFT/MRCI approach^15,16^ has been successfully used to describe polyenes^17^ and (keto)carotenoids^18−20^ but always within a static description as gradients are not available for such a method. A more promising strategy is to select semiempirical (SE) Hamiltonians and combine them with configuration interaction descriptions (SECI).^21−23^ In this case, however, another limit applies, namely, the intrinsic accuracy of the SE Hamiltonian.

While many previous theoretical studies have investigated polyenes of increasing length,^12,17,24−32^ much less has appeared to date for their analogues containing carbonyl (or hydroxy) groups.^18,20,33,34^ Hence, the first part of the present study is an effort to fill this gap by establishing reliable reference *ab initio* data for a set of model keto-carotenoids of *C*_2*h*_ symmetry and C_2*n*–2_H_4*n*–2_O_2_ stoichiometry, where *n* = 2–13 (from now on indicated as CO_*n*_). These reference data were used to assess the accuracy and reliability of TD-DFT and SECI approaches. In the second part of the study we have selected a representative example of the CO_*n*_ systems and analyzed the changes in nature and relative energy for its manifold of states due to structural deformations breaking the *C*_2*h*_ symmetry. Once again, we have compared *ab initio* methods with TD-DFT and SECI approaches. Finally, in the third part of the study, we have applied the SECI approach tested and established in the first two steps to investigate the orange carotenoid protein (OCP), a protein involved in cyanobacteria photoprotective mechanisms. Upon absorption of blue light, the xanthophyll embedded within the two domains of the protein (canthaxantin, CAN; see Scheme 1) translocates inside one domain and the protein opens up, thus allowing the interaction with the cyanobacterial antenna, the phycobilisome.^35,36^ In the past few years, many aspects of the opening process have been clarified,^37−39^ but the molecular details of the initial photochemical process, which takes place within a few picoseconds after the excitation of CAN, remain elusive.

**Scheme 1:**
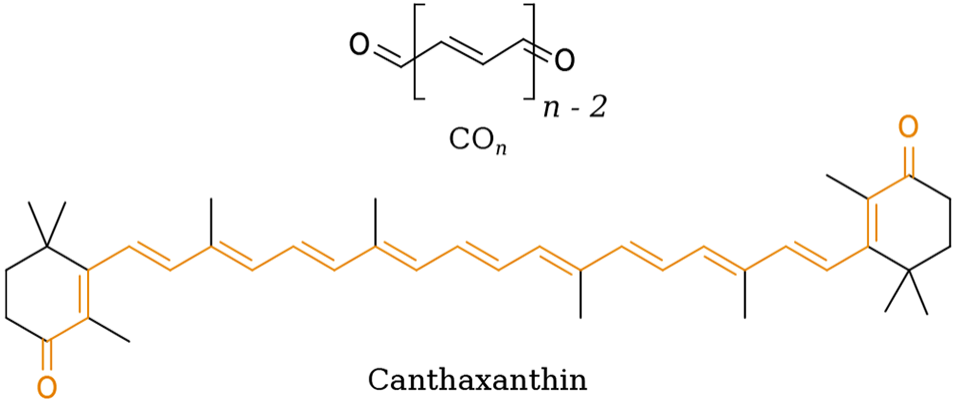
Chemical Structure of Model Keto-carotenoids (CO_*n*_) (top) and Structure of Canthaxantin with the CO_13_ Core Highlighted in Orange (bottom)

Because at present almost no routinely available methods are able to accurately and simultaneously describe static and dynamic correlation on systems of moderate size, we built our first part of the analysis on the comparison of a high-quality single-reference method and a multireference approach. For the former we selected the third-order coupled-cluster (CC3) and its equation of motion (EOM) extension to excited states^40,41^ (from now one we simply use CC3 for both ground and excited, EOM-CC3, calculations). As a multireference approach we selected CAS-SCF, and we constructed the “natural active space” of each CO_*n*_ characterized by *n* double bonds, in terms of *n* occupied and *n* virtual π orbitals, and the two occupied nonbonding lone-pair orbitals. Because the CAS-SCF method is strongly limited by the exponential scaling in the size of the active space, we decided to overcome this limitation by exploiting the advantages offered by density matrix renormalization group (DMRG).^42,43^ This method is able to provide approximate results for larger active spaces, and it was shown to be particularly well-suited for describing linear polyenes and carotenoids.^32,34,44^ To account also for the effects of dynamic correlation, we have applied a perturbative correction (NEVPT2) to DMRG-SCF energies.^45^ We note that DMRG/NEVPT2 has been successfully used to capture the effects of nuclear relaxation on the manifold of excited states for five extended carotenoids.^34^

Our analysis starts in a classical Franck–Condon (FC) framework by studying the excitation energies of low-lying electronic states of CO_*n*_ systems at their ground-state (GS) equilibrium geometries. However, obtaining reliable GS geometries for large π-conjugated systems is not trivial because a treatment of electronic correlation is needed to recover the correct degree of delocalization along the conjugated chain. Specifically, it is well-known that the bond length alternation (BLA), one of the most important geometrical parameters quantifying the degree of localization of the obtained electronic structure, is significantly dependent on the level of theory used.^28,46^ Because of its diagnostic importance in these systems we choose the BLA as our main reference parameter in selecting a GS optimization method. Because the GS of polyenes is a single reference state, we have taken as gold standard CC3, computing optimized geometries for systems with 2–6 double bonds (CO_2_–CO_6_) and comparing them to methods that allow treatment of larger systems, namely MP2, DFT/B3LYP,^47,48^ and DFT/CAM-B3LYP.^49^

To test the quality of the MP2 structures, we compared them with the CC3 ones for the shorter CO_*n*_ chains. The results, reported in Figure S1, show that the excitation energies calculated with the two sets of structure are extremely similar. The results reported in Figure S2 clearly show that CAM-B3LYP gives a too overlocalized picture of the conjugated system as compared to CC3, while B3LYP exaggerates the system conjugation. For longer systems instead, B3LYP behaves similarly to MP2; such behaviors are consistent with previous studies on other conjugated systems.^28,46^ On the basis of this analysis, we selected MP2 as the method of choice for the optimization of the ground-state geometries of CO_*n*_ compounds with up to 13 double bonds.

To characterize the excited states, we calculated the electron density changes upon electronic excitation for each different state of the CO_13_ system at the DMRG-SCF level. The results are shown in Figure 1. It is clear that the *B*_*u*_^+^, *B*_*u*_^–^, and *A*_*g*_ states share a similar density displacement in which π density is reduced on the double bonds and increased on the single ones. We also note, from the apparently different volumes embraced by the iso-density surfaces, that the density reorganization is larger for the dark states (*B*_*u*_^–^ and *A*_*g*_) than for the bright one (*B*_*u*_^+^), consistent with the better description of the latter with single-reference methods. In contrast, the density differences of both the *A*_*u*_ and *B*_*g*_ states confirm their n → π* nature, in which the density is displaced from the terminal regions corresponding to lone pairs of the oxygen atoms to the π* orbitals vicinal to the carbonyl group. The fact that n → π* transitions, which are nearly degenerate in longer systems such as CO_13_, involve both carbonyl groups is a consequence of the imposed symmetry, and they can be more conveniently represented as localized transitions on each of the two carbonyl groups.

**Figure 1:**
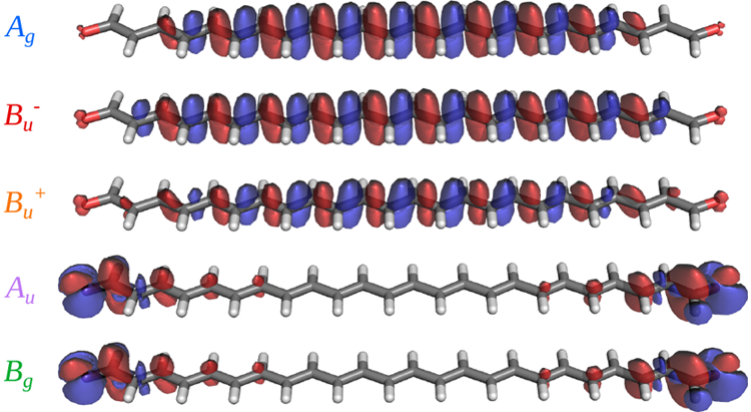
DMRG-SCF density difference between excited and ground state of CO_13_ represented as Δρ (Δρ = ρ_*n*_ – ρ_GS_) isosurfaces at +0.001 (red) and −0.001 (blue).

More detailed analyses of the DMRG wave functions were performed by using the mutual information diagrams^50,51^ (see Figure S3) and by estimating the contribution of single Slater determinants in the wave function^52^ (see Table S3). The latter scheme allows for a more straightforward interpretation: one can easily recognize that the GS has a largely dominant HF character, while the two n → π* transitions are a combination of single excitations from one nonbonding orbital to π* orbitals. In contrast, the dark π → π* transitions (*A*_*g*_ and *B*_*u*_^–^) show large contributions of both double and single excited determinants, while the bright *B*_*u*_^+^ state has a predominant HOMO → LUMO character without any significant contribution of double excited determinants. This confirms what was expected, namely, that the *A*_*g*_ and *B*_*u*_^–^ states are the ones with the most important multireference character.

Let us proceed with the analysis of the transition energies by comparing CC3 with DMRG-SCF (Figure 2A) and DMRG-SCF/NEVPT2 (Figure 2B) results. Because the computational demand of CC3 is much larger than the one of DMRG, we limited the calculations performed with the former method to CO_8_, which is already a computational challenge.

**Figure 2:**
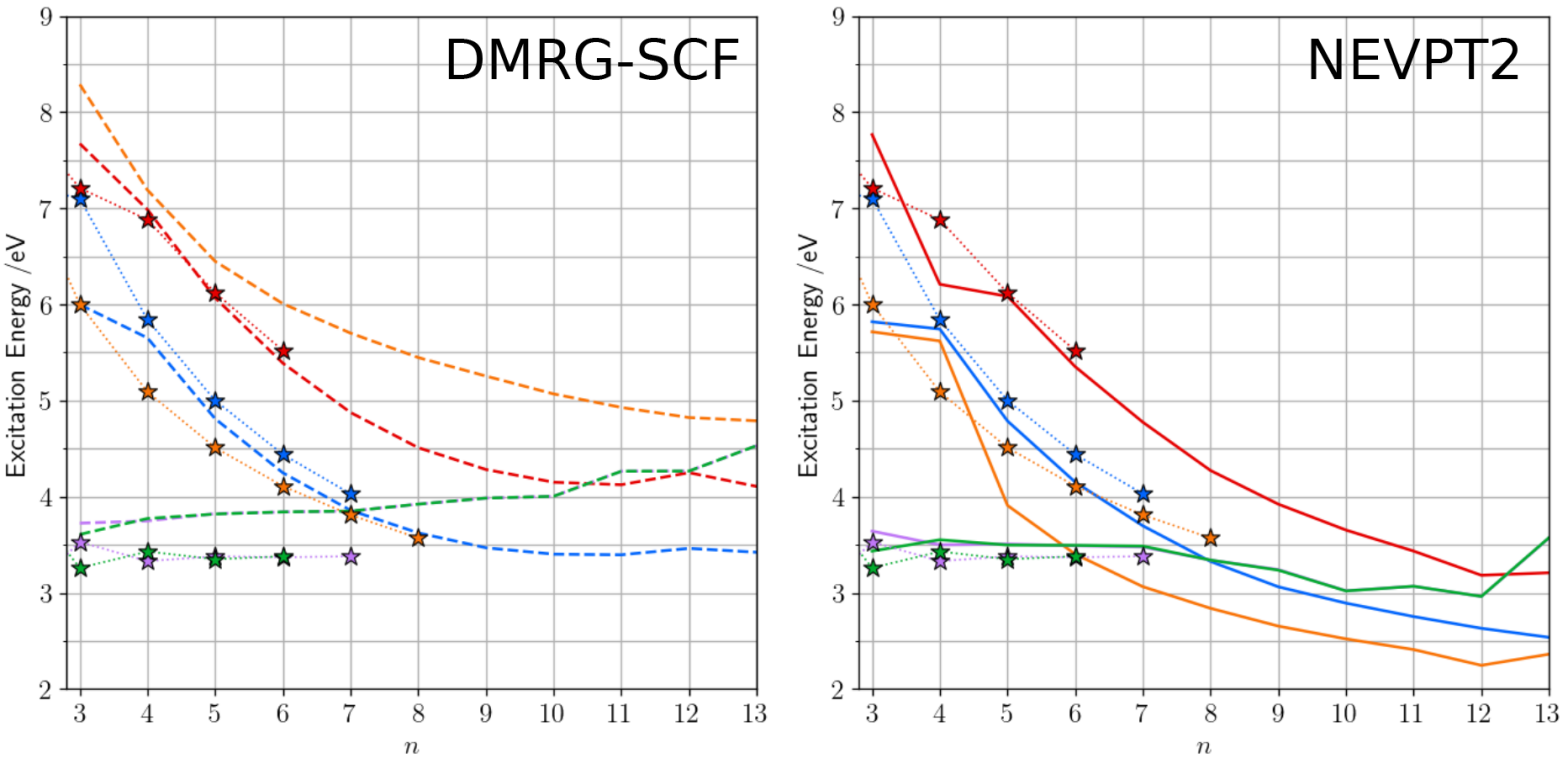
Excitation energies of CO_*n*_ systems at the DMRG-SCF level without (left panel) and with (right panel) NEVPT2 correction. CC3 data (stars) are also reported for the shorter systems. The color code indicates the electronic state: *A*_*g*_ in blue, *B*_*u*_^+^ in orange, *B*_*u*_^–^ in red, *B*_*g*_ in green, and *A*_*u*_ in violet. All calculations have been performed within a *C*_2*h*_ symmetry.

The comparison between CC3 and DMRG-SCF highlights advantages and drawbacks of these two methods, which were only partially predictable. In particular, we note a good agreement between the two for the dark *B*_*u*_^–^ state, but also for the dark *A*_*g*_ state, both being characterized by significant contributions from double excitations. At this stage, we recall that CC3 was shown to slightly overestimate the excitation energies of the dark *A*_*g*_ state of butadiene and hexatriene, but to be rather competitive with multireference methods,^11^ for these short systems in which the contributions of the doubles in the *A*_*g*_ state remain limited to *ca.* 25%. As we expect the double excitation character to increase with chain length, the CC3 error should become larger as longer systems are considered. The n → π* transitions are somehow blue-shifted in DMRG-SCF with respect to CC3, but it is the bright *B*_*u*_^+^ state which, quite surprisingly, shows the most significant difference. As the *B*_*u*_^+^ state is mainly characterized by single excitations, the corresponding excitation energy is expected to be accurately described by CC3; this implies that it is largely overestimated by DMRG-SCF likely because of the lack of dynamic correlation.

In order to improve these results we applied NEVPT2, in its state-specific (SS) formalism, on top of the DMRG-SCF. Looking at the NEVPT2-corrected results we note only minor changes for both dark π → π* states. In contrast, the transition energies are now in very good agreement with the CC3 ones for the two n → π* transitions. Two anomalies appear in the data: both the π → π* states of CO_4_ and the n → π* of CO_8_–CO_12_ correspond to a quasi degenerate situation (of *B*_*u*_^+^/*B*_*u*_^–^ and *B*_*u*_^–^/*A*_*u*_/*B*_*g*_, respectively) in the multireference wave function (see Figure 2A). In these situations, SS-NEVPT2 is expected to show its shortcomings, which instead could likely be circumvented by quasi-degenerate (QD) NEVPT2.^53^

In any case, when the near-degeneracy effects become smaller (CO_13_), a smooth trend is recovered. The largest effect of the NEVPT2 correction is found for the bright *B*_*u*_^+^ state, which is significantly red-shifted with respect to DMRG-SCF, resulting in excitation energies which are 0.5–0.7 eV lower than the ones predicted with CC3. We interpret this outcome as an overcorrection typical of second-order perturbative models when the starting point is far from the spot.

Combining all these considerations, we can conclude that DMRG/NEVPT2 is a good reference for the dark *A*_*g*_ and *B*_*u*_^–^ states; the same holding for the n → π* states as far as they are well-separated (energetically) from other states. In contrast, one has likely to be cautious in considering this method as a reliable reference for the bright *B*_*u*_^+^ state as it is here shown that a significant underestimation of its excitation energy is systematically observed for the treated systems.

On the basis of this analysis, we have built a data set of benchmark excitation energies for the CO_*n*_ systems by mixing the most accurate results obtained with different methods. In this data set, we used (i) for *A*_*u*_ and *B*_*g*_, CC3 for short systems and NEVPT2 for longer ones; (ii) for *A*_*g*_, NEVPT2; (iii) for the bright *B*_*u*_^+^, CC3 for shorter systems and their extrapolation for longer systems. Details on the extrapolation procedure and numerical data of extrapolated/interpolated excitation energies (Table S2) are reported in the Supporting Information.

This data set can now be used to assess the performances of both TD-DFT (using B3LYP and CAM-B3LYP functionals) and SECI methods. The results for the FC excitation energies of the CO_*n*_ systems are reported in Figure 3. We note that SECI results are reported for CO_*n*_ systems with *n* > 4 because of the low accuracy of SECI to describe the shortest systems.

**Figure 3:**
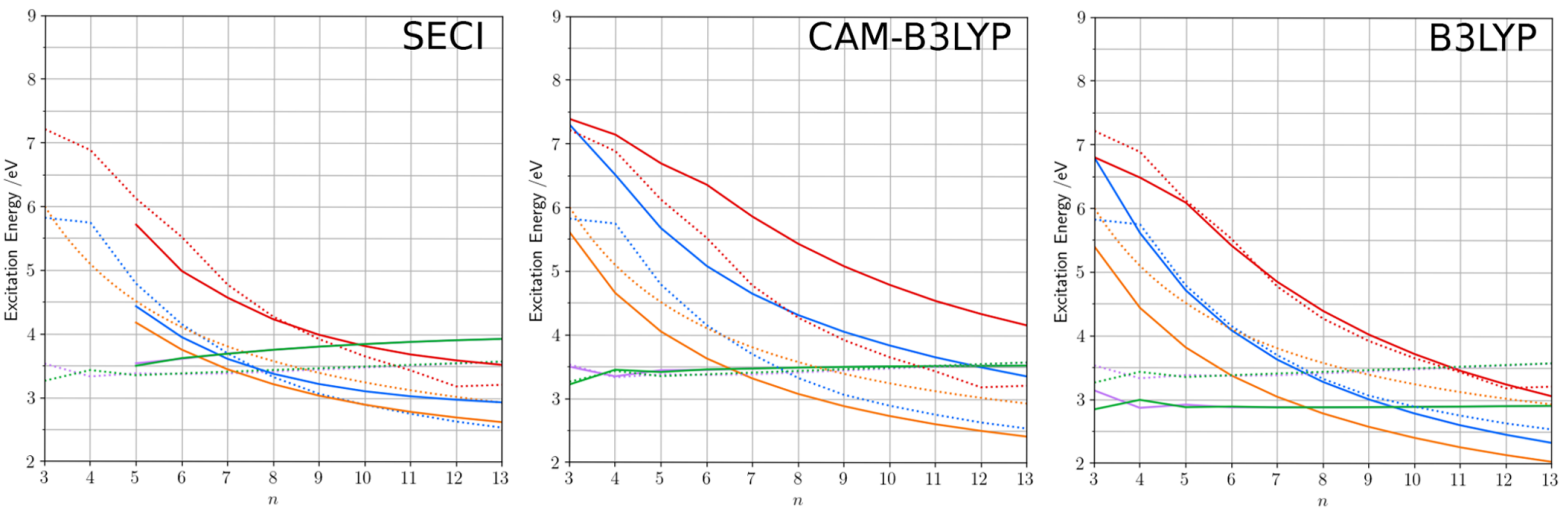
Comparison of SECI (left panel), TD-CAM-B3LYP (central panel), and TD-B3LYP (right panel) excitation energies of CO_*n*_ systems with the benchmark values obtained from CC3 and DMRG-SCF/NEVPT2 (dotted lines). The same color code is used as in Figure 2 (*A*_*g*_ in blue, *B*_*u*_^+^ in orange, *B*_*u*_^–^ in red, *B*_*g*_ in green, and *A*_*u*_ in violet).

Both TD-DFT and SECI give qualitatively good results, but quantitatively they significantly differ. Focusing on TD-DFT, one notes that both the tested functionals give the same ordering of the states with the n → π* transitions being the lowest ones for the short systems (*n* = 4–6(7)) and the bright *B*_*u*_^+^ state becoming the lowest for longer chains. However, the two functionals behave quite differently when quantitatively compared with the benchmark data. In more detail, CAM-B3LYP gives very accurate results for the n → π* energies while it significantly overestimates the dark π → π* energies (*A*_*g*_ and *B*_*u*_^–^) and underestimates the transition energy to the bright *B*_*u*_^+^ state. Unexpectedly, B3LYP seems to give a much more faithful picture than CAM-B3LYP even for the *A*_*g*_ state—despite its significant double excitation character—as well as for the other dark *B*_*u*_^–^ state. For the other states, generally underestimated energies (by about 0.5 eV) are found with this functional. Comparisons of the transition densities computed at TD-B3LYP and DMRG-SCF levels for CO_13_ indeed show that such an agreement on excitation energies correspond to a qualitative agreement on the description of the underlying electronic states (see Figure S5).

The picture offered by SECI is somehow more balanced than the one obtained with TD-DFT. Interestingly, the energies computed with this method are within 0.5 eV from the reference values for all the states of all the systems. It should also be recalled that the SECI calculations on a medium sized system (CO_11_) are about 3 orders of magnitude cheaper (computationally) than their TD-DFT counterpart which are in turn 2 orders of magnitude cheaper than the DMRG-SCF calculations.

The next step in our investigation is to move away from the FC region and to introduce structural deformations lifting the *C*_2*h*_ symmetry. To this end, we focused on a representative system (CO_11_) and selected a few degrees of freedom that, from chemical intuition, should play a role in the relaxation of the excited states after the Franck–Condon excitation: the BLA and torsions around a single and a double bond in the terminal part of the chain (quantified by the ϕ_2_ and θ_2_ dihedral angles, respectively; see Figure 4).

**Figure 4:**
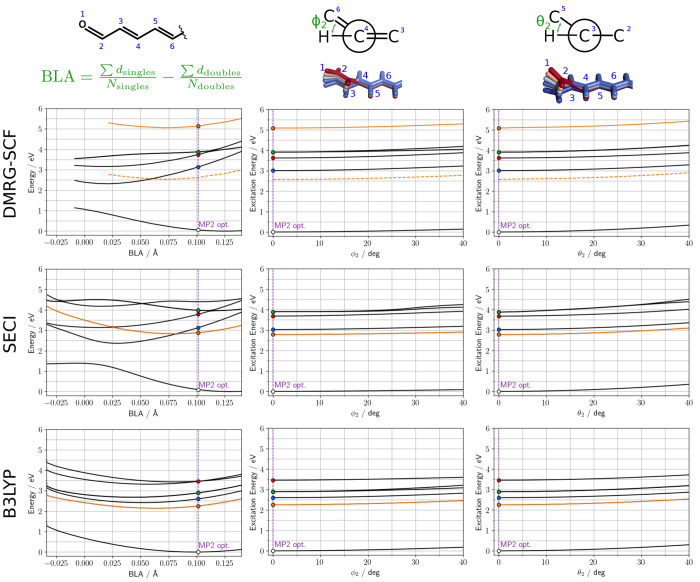
Potential energy surfaces of CO_11_ along key degrees of freedom computed with different methods (from top to bottom: DMRG-SCF, SECI, and TD-DFT/B3LYP). From left to right: BLA, ϕ_2_ (distortion of the second C–C single bond, 0 = *s*-trans, 180 = *s*-cis), θ_2_ (distortion of the second double bond (C=O being the first one), 0 = trans, 180 = cis). The point corresponding to the fully optimized MP2 geometry is indicated by a vertical dashed line, and colored circles indicate the electronic state according to the same color code used in the previous figures. The state with the largest transition dipole is always in orange; for DMRG, an additional curve has been reported (dashed line), obtained by shifting the energies by the magnitude of the NEVPT2 correction determined at the GS equilibrium geometry.

For each of these degrees of freedom we performed relaxed MP2 scans and computed excitation energies with DMRG-SCF, SECI, and TD-B3LYP. The results of these calculations are reported in Figure 4. Because we have already noted that the main pitfall of DMRG-SCF is to severely overestimate the energy of the bright *B*_*u*_^+^ state, we reported both its original position and the one shifted by the magnitude of the NEVPT2 correction determined at the GS equilibrium geometry.

Let us start by considering the distortion of the ϕ_2_ and θ_2_ dihedral angles. All three methods considered (DMRG-SCF, SECI, and TD-B3LYP) yield qualitatively similar results. Even though they provide a different ordering of the electronic states, they all predict that the distortion of either ϕ_2_ or θ_2_ increases the energy of all the electronic states. Moreover, all methods agree that the rotation around the single bond (ϕ_2_) lifts the quasi-degeneracy of the two n → π* transitions after *ca.* 20°, while twisting the double bond (θ_2_) leaves the two transitions almost degenerate even at quite large dihedral angles. All these predictions fit chemical intuition: the distortion of a dihedral angle is expected to reduce the “effective conjugation length” causing an upshift of all the electronic levels in the molecule. Moreover, while the rotation around the single bond is expected to generate two independent π systems, each of them with its own set of orbitals and therefore with different energies for the n → π* transition, the rotation of the double bond should induce a deformation of the π orbitals without isolating the two sides of the system, thus leaving the two n → π* nearly degenerate.

When considering the dependence on the BLA, a much more complex profile is found, and qualitative differences are noticed between the different methods. At the DMRG-SCF level, the π → π* states calculated at the BLA coordinate in the FC region are far from their minima. Indeed, if the BLA in the GS minimum is 0.125 Å, its *A*_*g*_ and *B*_*u*_^–^ counterparts are *ca.* 0.025 Å, whereas the *B*_*u*_^+^ state is most stable for a BLA of 0.075 Å. All these states show roughly harmonic surfaces in the BLA space. In contrast, the GS presents a large anharmonicity for BLA smaller than 0.025 with a plateau behavior. Unfortunately, we were not able to obtain a fully converged DMRG-SCF wave function for any structure with BLA lower than −0.009 Å, making an *ab initio* analysis of this part of the PES impossible. Considering now the interplay between the different states, once the energetic position of the *B*_*u*_^+^ is corrected, it seems that upon excitation, this state should relax along the BLA coordinate through a conical intersection with the *A*_*g*_ state. We note that this conversion should be rather fast because, according to our results, it does not involve large motions. Such description is consistent with the experimental observations.^54^ We also note that the n → π* transitions are weakly influenced by the BLA and do not significantly couple with the other electronic states.

Once more, SECI is surprisingly good in reproducing all the relevant features of the DMRG-SCF PESs. The largest differences are seen for the n → π* states for which the energy slightly increases when reducing the BLA. Furthermore, SECI seems to be more numerically stable and data can be obtained even for BLA smaller than −0.025 Å. On the other hand, TD-DFT yields a very different picture with respect to DMRG-SCF, underlying the difficulties of this method in describing photochemically important features in carotenoids. In particular, it gives a completely harmonic picture of the GS energy which does not fit the trend produced by multireference methods. Indeed, this could be seen as an artificial increase of the GS energy at low BLA due to the lack of multireference effects in DFT. It is noteworthy that a similar trend is obtained with SE-HF (see Figure S6A). Moreover, all the excited states computed with TD-DFT show a very similar harmonic behavior with minima placed roughly at BLA of 0.050 Å.

To better understand the changes in the nature of the electronic states during the evolution on the BLA coordinate, we relied on the SECI description and compared the contributions of the different determinants in the wave function (see Figure S7). From this analysis, it is clear that, at the GS equilibrium geometry, the description provided by SECI is consistent with the one provided by DMRG-SCF for all the π → π* transitions (see Table S3). However, moving to smaller BLAs, a dramatic change in the character of the states takes place. In particular, we observe that the GS acquires a significant multireference character gaining an important contribution from the HOMO^2^ → LUMO^2^ determinant while simultaneously the *A*_*g*_ state gains an important contribution from the HF determinant, suggesting a mixing of those two states in proximity of their avoided crossing (BLA = 0.02 Å see Figure 4). The character of the *B*_*u*_^–^ state, instead, is only slightly influenced by the BLA, excluding the point close to the conical intersection with the *B*_*u*_^+^ state (BLA = 0.025 Å). Finally, the *B*_*u*_^+^ state shows a significant increase in the double excitation contribution at low BLA together with a loss of its “pure” HOMO → LUMO character. Because at low BLA values the GS presents a non-negligible multireference character, it is expected that the quality of MP2 geometries deteriorates moving away from the minimum. Therefore, we have compared SECI energies computed on BLA relaxed scans performed at MP2 and SECI levels (Figure S6B). From such a comparison, it is clear that the two methods show a good agreement in the BLA region between 0.025 and 0.130 Å, whereas, at lower BLA, the geometries obtained with two methods differ significantly, making the results in this region less trustworthy.

To conclude the analysis on CO_11_, we have performed SECI geometry optimizations of the ground and the two lowest excited states. The obtained values of BLA are indeed very close to those corresponding to the minima of each PES reported in Figure 4 (see Figure S6C). These results indicate that computing excited-state energies using a GS BLA scan is a reliable tool to investigate the excited-state PESs.

As a last step in our analysis, we investigated canthaxantin (CAN, see Scheme 1) in vacuum and within a biological matrix, namely, the orange carotenoid protein (OCP).

As above, we have performed a relaxed scan along the BLA coordinate of CAN at the MP2 level, but this time the optimizations have been repeated both in vacuum and in the protein using the ONIOM(QM:MM)^55^ model (see details in the Supporting Information). The resulting geometries were finally used to compute SECI excitation energies. To disentangle the effects induced on the geometry of CAN by the constrains of the protein binding pocket from the direct electrostatic effects on the excitation energies, the geometries obtained in the protein were also used to compute vacuum excitation energies. In Figure 5 we report the energy profiles calculated on the geometries optimized in the protein while the corresponding ones for the vacuum geometries can be found in the Supporting Information (Figure S8).

**Figure 5:**
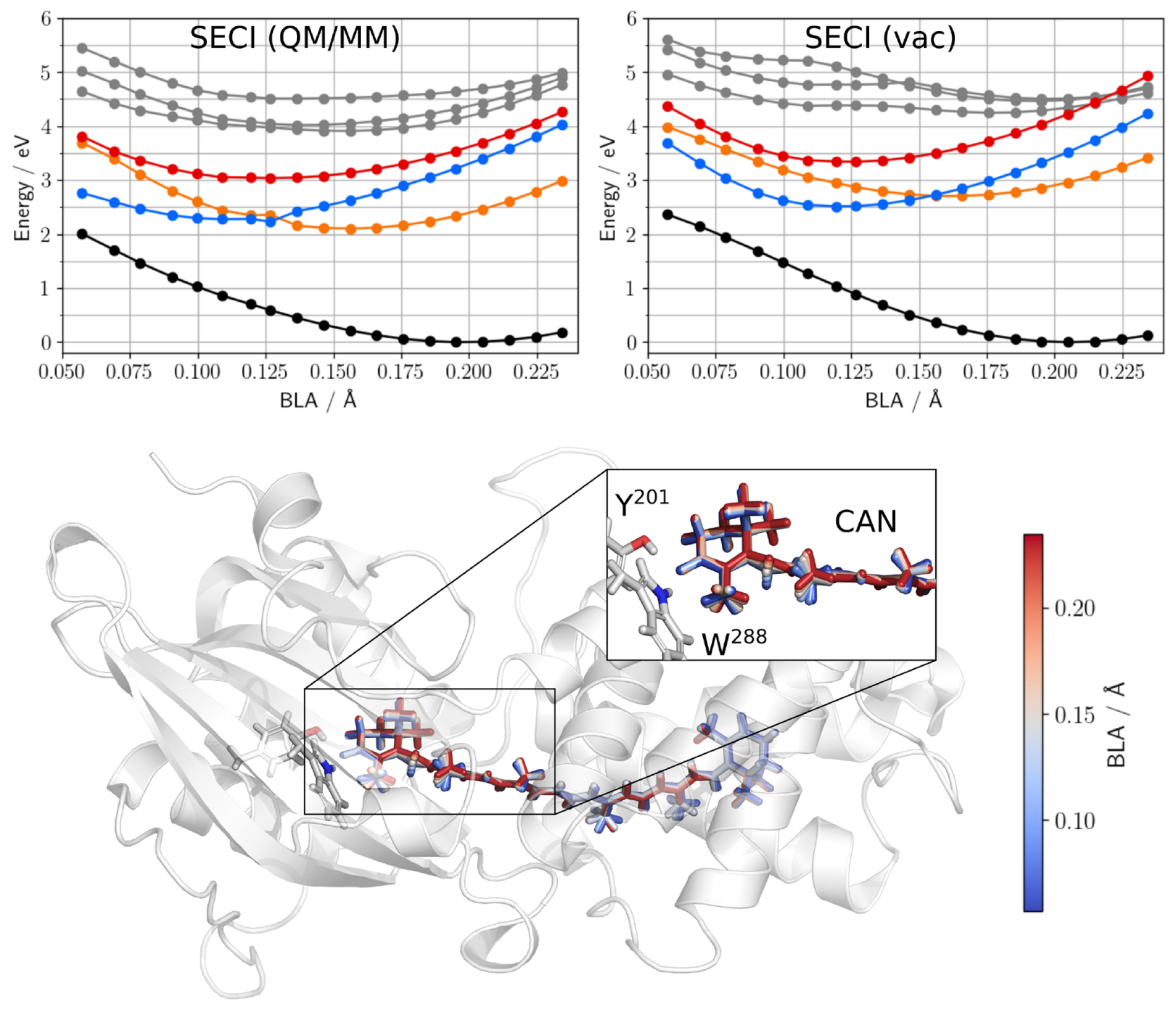
SECI energies of different electronic states of CAN calculated for different BLA values in OCP (QM/MM) and in vacuum: GS in black, *A*_*g*_ in blue, *B*_*u*_^+^ in orange, *B*_*u*_^–^ in red, and unidentified states in gray. In both cases the geometries are those optimized in OCP. Bottom panel: BLA value mapped on the color of CAN which is represented as licorice, together with the two H-bonding residues Y^201^ and W^288^.

From the comparison of Figures S8 and 4, we can clearly see that the energy profiles obtained for CAN outside its biological environment closely resemble the ones of the CO_11_ model system, the only key difference being that the GS minimum is found at a higher BLA. This can be explained upon recalling that CAN presents methyl groups on the two terminal rings which prevent a full planarization of the conjugated backbone.

When moving to the geometries optimized within the protein but still neglecting the effect of the protein electric field on the energies, one notices that the GS basin is much larger than the one obtained on vacuum geometries, and larger decreases of the BLA can be reached at a smaller energetic cost. This behavior is most likely a consequence of the constraints imposed by the protein residues in the binding pocket as confirmed by the fact that the most significant changes in the CAN structure along the BLA scan take place where it is hydrogen bonded with a tyrosin (Y^201^) and tryptophan (W^288^) residue (see Figure 5). Here, when BLA increases, the backbone of CAN tends to become more coplanar with the terminal ring. These findings show that, while in isolated model systems the BLA can be considered as a degree of freedom fully independent of other important structural parameters such as dihedral angles, this assumption does not hold for CAN in OCP. In the latter case, the excited-state relaxation likely happens on a space with a higher dimensionality which cannot be easily identified using chemical intuition only.

Moving now to the electrostatic embedding SECI/MM calculations of CAN in protein, we observe less steep profiles with respect to the ones calculated neglecting the effects of the protein (vac). As a result, the position of the conical intersection between the *A*_*g*_ and *B*_*u*_^+^ states is shifted to significantly smaller BLAs. Moreover, in the region of very low BLA (below 0.10 Å), the protein electric field strongly affects the behaviors of both the GS and *A*_*g*_ states, bringing them at least 0.25 eV closer to each other than in vacuum.

All these observations together suggest that the excited-state dynamics of CAN in OCP is significantly different than in vacuum as the protein plays an important role at two levels. First, the constraints induced by the protein binding pocket break the symmetry of CAN, thus enabling new relaxation pathways. Second, the protein electric field induces a further fine-tuning of the electronic structure of the carotenoid affecting the relaxation pathways. From these results, we propose some insights on the photoactivation mechanism of the OCP complex. First, it is clear that after the vertical excitation to the *B*_*u*_^+^ state, CAN relaxes with a significant reduction of its BLA. As already commented, it is likely that this major structural change is accompanied by changes in dihedral angles and other internal coordinates. Along this relaxation pathway, CAN rapidly reaches the conical intersection between the *B*_*u*_^+^ and *A*_*g*_ states allowing a population transfer. The final step is expected to be the relaxation from *A*_*g*_ to the GS. From the present comparison of the QM/MM and vacuum energy profiles, it appears that the protein strongly reduces the gap between the two states. Even if this effect should facilitate the relaxation to a high-energy region of the GS PES (coherently with experimental observations^56^), unfortunately from our data we cannot determine whether the population of a metastable “hot” GS, which could resemble the S* state, is possible. The confirmation of such a hypothesis in fact requires considering the coupling of the BLA with other structural degrees of freedom, a type of analysis that can be done only with nonadiabatic dynamics techniques.

In the present Letter, for the first time we have compared state-of-the-art *ab initio* methods and computationally cheaper ones on the manifold of electronic states of keto-carotenoids of increasing conjugation length, giving a comprehensive view of advantages and drawbacks of each method. From such a comparison we found that SECI is by far the most reasonable choice as it gives a balanced picture of all the low-lying states both in proximity of the FC point and along relevant relaxation coordinates. On the other hand, we showed that DMRG-SCF tends to overestimate the excitation energies, in particular for the bright *B*_*u*_^+^ state. Moreover, such an overestimation cannot be satisfactorily corrected using DMRG/NEVPT2. We also confirmed that the widely used TD-DFT reasonably behaves near the FC point, but it dramatically fails when the multireference character of the states increase (e.g., at low BLA values), making it useless to study the photochemistry. We finally applied SECI to investigate cantaxanthin in the orange carotenoid protein, showing the strong impact that the protein matrix has on the electronic states through geometry constraints imposed by the binding pocket and by electric field effects.

The study has been focused on one type of xanthophylls (those containing conjugated keto groups), but we are confident that the main conclusions drawn here are general. In fact, xanthophylls that do not present keto groups can be seen as subcases of the keto systems. Our data, in fact, show that for long systems the “additional” n-π* states are high in energy and that they do not significantly couple with the low-lying π–π* states. From our study we can thus conclude that SECI is an effective computational strategy for describing excited states of xanthophylls, and we expect that, when applied to xanthophyll containing proteins, it will help to answer the still open questions on their function. The still missing piece is the integration of such a strategy with a nonadiabatic dynamics approach; an extension of SECI/MM to surface hopping simulations is already in progress in our group.

## Supporting Information Available

Methodological details on computational methods used; numerical values of CO_*n*_ excitation energies at different levels of theory; details on the fitting procedure performed on *B*_*u*_^+^ CC3 excitation energies; numerical values of interpolated and fitted excitation energies of CO_*n*_; MP2 optimized structures of *C*_2*h*_ CO_*n*_ (*n* = 2–13); comparison of excitation energies computed on CC3 and MP2 optimized structures; comparison of BLA computed with different methods; decomposition of DMRG-SCF wave functions of CO_13_ in Slater determinants; mutual information diagrams for DMRG-SCF wave functions of CO_13_; converged DMRG-SCF active space molecular orbitals of CO_13_; transition densities of CO_13_ computed at DMRG-SCF level and at TD-B3LYP level; comparison of GS of CO_11_ at different values of BLA computed at SECI level and at semiempirical HF level; comparison of SECI excitation energies of CO_11_ at different values of BLA computed on MP2 and SECI optimized geometries; decomposition in Slater determinants of SECI wave functions of CO_11_ at different values of BLA; SECI excitation energies for CAN at different values of BLA; values of BLA for optimized C_11_ geometries on SECI ground and excited-state PESs; DMRG-CI excitation energies for CO_13_ computed with different bond lengths (PDF)
