# Supplementary material for: Excited States of Xanthophylls Revisited: Toward the Simulation of Biologically Relevant Systems
Source: J Phys Chem Lett. 2021 Jul 12;12(28):6604–12. doi: 10.1021/acs.jpclett.1c01929 (PMC8311646; doi:10.1021/acs.jpclett.1c01929)
Supplement: Supplementary file 1 — jz1c01929_si_001.pdf [file jz1c01929_si_001.pdf]

Supporting Information: Excited States of Xanthophylls Revisited: Towards the Simulation of Biologically Relevant Systems

Mattia Bondanza,*,† Denis Jacquemin,‡ and Benedetta Mennucci*,†

†*Dipartimento di Chimica e Chimica Industriale, University of Pisa, via G. Moruzzi 13, 56124, Pisa, Italy*

‡*Université de Nantes, CNRS, CEISAM UMR 6230, F-44000 Nantes, France*

E-mail: mattia.bondanza@phd.unipi.it; benedetta.mennucci@unipi.it

S1 Methods

Coupled Cluster Calculations

The CC3 and EOM-CC3[S1,S2] calculations have been performed with the Cfour package.[S3] From now on we simply use CC3 for both ground and excited (EOM-CC3) calculations. We used the frozen-core approximation and the *aug*-cc-pVDZ atomic basis set for these calculations that were made considering the point group symmetry for obvious computational reasons. It is well-recognized that CC3 approach typically yields highly-accurate transition energies, on average within ca. 0.05 eV of the FCI limit.[S4] However, as all single-reference method, CC3 has difficulty tackling transitions with a multi excitation character. More in details, one needs to distinguish the transitions that are "purely double" for which CC3 miserably fails, to those that have a significant, yet not dominant, double-excitation character, such as the A_g transition in butadiene and hexatriene, for which CC3 slightly overshoots the transition energies.[S5] For the latter category, CC3 seems in fact to provide an accuracy similar to the one obtained with CAS-PT2 or NEV-PT2.[S5]

DMRG(CAS)-SCF/NEVPT2 Calculations

DMRG-SCF calculations have been performed with in-house scripts based on the most recent version of PySCF[S6,S7] which is able to perform CAS-SCF with orbital optimization decoupled from the Full Configuration Interaction (FCI) problem.[S8] Within this setup one can use, in a modular way, approximate FCI solvers such as Quantum Chemical Density Matrix Renormalization Group (DMRG for short) or Quantum Monte Carlo (QMC) while performing a CAS-SCF like orbital optimization. All the CAS-CI calculation performed in this work have been performed using the "*natural active space*" for CO_n system which include n π orbitals, n π^* orbitals and 2 non bonding orbitals (located on the carbonyls' oxygen atoms) with $2n + 2$ electrons. This choice leads to active spaces that are too large to be tractable with standard FCI solvers for systems larger than CO_6. To overcome this limit

we used the DMRG solver for all the active spaces containing more than 16 active orbitals. DMRG calculations have been performed using StackBlock 1.5 coupled to the PySCF interface.[S9] All DMRG calculations have been performed with a maximum bond length (m) of 2000; default StackBlock 1.5 parameters have been used for orbital ordering and initial guess for the MPS wave function. The initial active space for the CAS-SCF optimization was selected exploiting the symmetries of canonical SCF orbitals when possible. In all the other cases, we applied a simple localization/selection procedure which is described below. The optimization of active orbitals was done using a State-Average (SA) CAS-SCF procedure with equal weights for all the states extracted from the CI; when symmetry is used, all the electronic state selected in different irreducible representations (*irrep*) have been included in the state averaging, therefore in our calculation also electronic states from with different *irrep* are orthogonal. In all calculations, we used the default convergence parameters provided by PySCF. Density fitting was used in all the calculations to reduce the cost associated to the dimension of the basis set.

Localization Procedure

DMRG-SCF calculations on structures that do not belong to the C_{2h} point group have been performed using a localized molecular orbital basis set. Such orbitals are obtained from canonical SCF ones with the following procedure: (a) occupied orbitals are localized following the intrinsic bond orbital procedure,[S10] (b) Localized Intrinsic Valence Virtual Orbitals (LIVVO)[S11] are then constructed, defining a hard-virtual subspace which (c) is processed as suggested by Subotnik *et al.*[S12] On the resulting set of orbital we selected the π-active space using the procedure proposed by Keal *et al.*:[S13] a local conjugation plane is defined on the basis of molecule's geometry and a "π-score", which is later used for the selection, is computed using the overlap of the localized molecular orbitals with an atomic basis set aligned to the conjugation plane. This procedure was implemented in PySCF framework; the rotated basis set was computed using the recursive equations by Ivanic *et*

al.[S14]

TD-DFT Calculations

TD-DFT calculations have been performed using Gaussian 16 software package.[S15] All of these calculations have been performed with the Gaussian 16 default parameters. Two different functionals have been tested, namely B3LYP,[S16,S17] and CAM-B3LYP[S18]).

Semi-Empirical Configuration Interaction Calculations

Semi-Empirical Configuration Interaction (SECI) calculations have been performed with the MNDO software package. The orbitals used for the CI expansion are computed using the OM2 Hamiltonian in a SCF calculation targeting an open-shell singlet state having two singly occupied orbitals. Then we constructed an active space with all the π, n, and π^* orbitals and included all single excitations from two reference determinants (the closed-shell HF singlet, and the doubly excited $\text{HOMO}^2 \rightarrow \text{LUMO}^2$). The selection of π-type orbitals have been done using the so-called π-population approach proposed by Thiel and coworkers.[S13]

Geometry Optimization

All the geometry optimizations but the CC3 ones have been performed using Gaussian 16 software package. For MP2 calculations we used 6-31G(p) basis set.

For scan along the BLA and dihedral angles coordinates, we defined those variables in the redundant coordinate set used in the optimization and performed a relaxed scan along the variable itself. This was achieved using the so-called Generalized Internal Coordinates (GIC) input implemented in Gaussian 16. The BLA definition used is the one reported in Figure 4 of the main text: it should be noted that since we include C=O bonds in the average of double bonds length the value reported in this paper are not directly comparable with BLA of polyenes. For the canthaxanthin optimization we computed the BLA on the conjugated

backbone highlighted in Scheme 1 of the main text.

Optimizations in the protein have been performed with the ONIOM(QM:MM) model[S19] as implemented in Gaussian 16; AMBER force field was used for the low-level calculations with the parameters used in previous works from some of us.[S20]

The CC3 optimization were performed with Cfour,[S3] using Z-matrix coordinates enforcing the point group symmetry and correlating all electrons. Default geometry convergence parameters were applied.

S2 Numerical Data and Procedures

Excitation Energies for symmetric CO_n

Table S1: Numerical data for the excitation energies of symmetric CO_n molecules. These energies are used in Figures 2 and 3 and are given in eV.

Method	n	A_g	B_u^-	B_u^+	A_u	B_g
CC3	2	7.27	8.05	7.59	2.85	4.15
	3	7.10	7.21	6.00	3.53	3.26
	4	5.84	6.88	5.09	3.33	3.43
	5	5.00	6.12	4.51	3.38	3.35
	6	4.44	5.52	4.11	3.37	3.38
	7	4.03	–	3.81	3.38	–
	8	–	–	3.57	–	–
DMRG	3	6.00	7.67	8.29	3.72	3.61
	4	5.65	6.99	7.19	3.75	3.77
	5	4.81	6.07	6.45	3.82	3.82
	6	4.25	5.39	6.01	3.84	3.84
	7	3.86	4.88	5.70	3.85	3.85
	8	3.62	4.51	5.45	3.92	3.92
	9	3.47	4.28	5.25	3.99	3.99
	10	3.40	4.15	5.07	4.00	4.00
	11	3.40	4.12	4.93	4.26	4.26
	12	3.46	4.25	4.82	4.27	4.27
	13	3.42	4.11	4.79	4.53	4.53
NEVPT2	3	5.82	7.77	5.72	3.64	3.43
	4	5.74	6.21	5.62	3.50	3.55

(Continued on next page)

Method	n	A_g	B_u^-	B_u^+	A_u	B_g
NEVPT2	5	4.79	6.08	3.91	3.51	3.50
	6	4.15	5.35	3.40	3.49	3.49
	7	3.70	4.77	3.06	3.48	3.49
	8	3.33	4.27	2.84	3.34	3.34
	9	3.06	3.92	2.65	3.24	3.24
	10	2.89	3.65	2.52	3.02	3.02
	11	2.75	3.43	2.41	3.07	3.07
	12	2.63	3.18	2.25	2.96	2.96
	13	2.54	3.21	2.36	3.57	3.57
SECI	3	7.82	–	6.05	3.37	3.09
	4	6.94	–	5.13	3.44	3.43
	5	4.43	5.71	4.18	3.54	3.50
	6	3.95	4.99	3.75	3.61	3.62
	7	3.61	4.57	3.45	3.69	3.69
	8	3.38	4.23	3.21	3.75	3.75
	9	3.22	3.99	3.03	3.80	3.80
	10	3.10	3.81	2.89	3.85	3.84
	11	3.03	3.68	2.78	3.88	3.88
	12	2.97	3.59	2.70	3.91	3.91
	13	2.93	3.52	2.62	3.93	3.93
CAM-B3LYP	3	7.29	7.38	5.61	3.50	3.22
	4	6.51	7.14	4.65	3.35	3.45
	5	5.67	6.69	4.05	3.44	3.41
	6	5.08	6.35	3.63	3.45	3.46

(Continued on next page)

Method	n	A_g	B_u^-	B_u^+	A_u	B_g
CAM-B3LYP	7	4.65	5.86	3.32	3.47	3.47
	8	4.31	5.43	3.08	3.49	3.49
	9	4.05	5.08	2.89	3.50	3.50
	10	3.84	4.79	2.73	3.51	3.51
	11	3.65	4.54	2.60	3.51	3.51
	12	3.50	4.33	2.50	3.52	3.52
	13	3.36	4.16	2.41	3.52	3.52
B3LYP	3	6.79	6.80	5.40	3.14	2.84
	4	5.61	6.48	4.44	2.87	2.99
	5	4.71	6.09	3.82	2.92	2.88
	6	4.09	5.41	3.38	2.88	2.89
	7	3.63	4.85	3.04	2.88	2.88
	8	3.28	4.39	2.78	2.88	2.88
	9	3.01	4.02	2.57	2.88	2.88
	10	2.79	3.72	2.40	2.89	2.89
	11	2.60	3.46	2.26	2.89	2.89
	12	2.45	3.24	2.13	2.90	2.90
	13	2.33	3.06	2.03	2.91	2.91

Fitting of B_u^+ CC3 Excitation Energies

To obtain a reference data set of B_u^+ excitation energies on CO_n compounds longer then CO_8 we used an extrapolation procedure. We performed a least-square fit of the excitation energies in eV computed for compounds CO_3 – CO_8 with the function:

$$E_{B_u^+}(n) = \frac{a}{n+b} + c. \tag{S1}$$

The optimized parameters are a=15.14348998, b=0.62407386 and c=1.81921892. The fitting yields a r^2 as large as 0.999992, confirming the regular evolution trend of the B_u^+ excitation energies with increasing chain length in CO_n oligomers.

Table S2: Numerical data for interpolated/fitted excitation energies of symmetric CO_n molecules. These energies are used in Figures 2 and 3 and are given in eV.

n	A_g	A_u	B_u^-	B_u^+	B_g
3	5.819385	3.530000	7.210000	5.997801	3.260000
4	5.743643	3.330000	6.880000	5.094143	3.430000
5	4.787411	3.380000	6.120000	4.511838	3.350000
6	4.152054	3.370000	5.520000	4.105348	3.380000
7	3.697005	3.380000	4.774625	3.805492	3.407794
8	3.326255	3.411695	4.272177	3.575174	3.435588
9	3.063144	3.443389	3.922754	3.392720	3.463382
10	2.893267	3.475084	3.653324	3.244613	3.491176
11	2.753416	3.506778	3.434339	3.121989	3.518970
12	2.631473	3.538473	3.183024	3.018791	3.546764
13	2.537187	3.570167	3.209075	2.930743	3.574558

MP2 C_{2h} Optimized Geometries of CO_n ($n = 2 - 13$)

All the geometries are provided in XYZ format; units are Å.

XYZ Geometry CO_2

```
C    +00.646037    +00.393159    +00.000000

H    +00.530088    +01.491855    +00.000000
```

C	-00.646037	-00.393159	+00.000000
H	-00.530088	-01.491855	+00.000000
O	-01.731161	+00.171537	+00.000000
O	+01.731161	-00.171537	+00.000000

XYZ Geometry CO_3

C	+00.584327	-00.333847	+00.000000
C	-00.584327	+00.333847	+00.000000
H	+00.620864	-01.422079	+00.000000
H	-00.620864	+01.422079	+00.000000
C	+01.876403	+00.372809	+00.000000
H	+01.816514	+01.479446	+00.000000
C	-01.876403	-00.372809	+00.000000
H	-01.816514	-01.479446	+00.000000
O	-02.957689	+00.205697	+00.000000
O	+02.957689	-00.205697	+00.000000

XYZ Geometry CO_4

C	+00.648517	+00.318648	+00.000000
C	-00.648517	-00.318648	+00.000000
C	+01.818929	-00.356580	+00.000000
C	-01.818929	+00.356580	+00.000000
H	+00.667947	+01.409704	+00.000000
H	-00.667947	-01.409704	+00.000000
H	+01.855561	-01.444083	+00.000000
H	-01.855561	+01.444083	+00.000000
C	+03.101096	+00.355183	+00.000000
H	+03.030303	+01.462810	+00.000000

C	-03.101096	-00.355183	+00.000000
H	-03.030303	-01.462810	+00.000000
O	-04.191765	+00.208088	+00.000000
O	+04.191765	-00.208088	+00.000000

XYZ Geometry CO_5

C	+00.585003	-00.343688	+00.000000
C	-00.585003	+00.343688	+00.000000
C	+01.876379	+00.295151	+00.000000
C	-01.876379	-00.295151	+00.000000
C	+03.055509	-00.368306	+00.000000
C	-03.055509	+00.368306	+00.000000
H	+00.562393	-01.433971	+00.000000
H	-00.562393	+01.433971	+00.000000
H	+01.890839	+01.387018	+00.000000
H	-01.890839	-01.387018	+00.000000
H	+03.103513	-01.455318	+00.000000
H	-03.103513	+01.455318	+00.000000
C	+04.326747	+00.357421	+00.000000
H	+04.240495	+01.464556	+00.000000
C	-04.326747	-00.357421	+00.000000
H	-04.240495	-01.464556	+00.000000
O	-05.426864	+00.188648	+00.000000
O	+05.426864	-00.188648	+00.000000

XYZ Geometry CO_6

C	+00.644985	+00.316119	+00.000000
C	-00.644985	-00.316119	+00.000000

C	+01.819387	-00.367537	+00.000000
C	-01.819387	+00.367537	+00.000000
C	+03.105688	+00.277089	+00.000000
C	-03.105688	-00.277089	+00.000000
C	+04.291400	-00.376360	+00.000000
C	-04.291400	+00.376360	+00.000000
H	+00.669689	+01.407090	+00.000000
H	-00.669689	-01.407090	+00.000000
H	+01.801030	-01.457884	+00.000000
H	-01.801030	+01.457884	+00.000000
H	+03.113288	+01.369288	+00.000000
H	-03.113288	-01.369288	+00.000000
H	+04.348734	-01.462899	+00.000000
H	-04.348734	+01.462899	+00.000000
C	+05.554624	+00.360452	+00.000000
H	+05.456841	+01.466934	+00.000000
C	-05.554624	-00.360452	+00.000000
H	-05.456841	-01.466934	+00.000000
O	-06.661295	+00.173094	+00.000000
O	+06.661295	-00.173094	+00.000000

XYZ Geometry CO_7

C	+00.588747	-00.341184	+00.000000
C	-00.588747	+00.341184	+00.000000
C	+01.874906	+00.294433	+00.000000
C	-01.874906	-00.294433	+00.000000
C	+03.054717	-00.381806	+00.000000
C	-03.054717	+00.381806	+00.000000

C	+04.335383	+00.271677	+00.000000
C	-04.335383	-00.271677	+00.000000
C	+05.527556	-00.370906	+00.000000
C	-05.527556	+00.370906	+00.000000
H	+00.566142	-01.432182	+00.000000
H	-00.566142	+01.432182	+00.000000
H	+01.894996	+01.385769	+00.000000
H	-01.894996	-01.385769	+00.000000
H	+03.043539	-01.472246	+00.000000
H	-03.043539	+01.472246	+00.000000
H	+04.334238	+01.364042	+00.000000
H	-04.334238	-01.364042	+00.000000
H	+05.594834	-01.456863	+00.000000
H	-05.594834	+01.456863	+00.000000
C	+06.782917	+00.377620	+00.000000
H	+06.673680	+01.483212	+00.000000
C	-06.782917	-00.377620	+00.000000
H	-06.673680	-01.483212	+00.000000
O	-07.895599	+00.143828	+00.000000
O	+07.895599	-00.143828	+00.000000

XYZ Geometry CO_8

C	+00.642437	+00.316905	+00.000000
C	-00.642437	-00.316905	+00.000000
C	+01.822629	-00.362901	+00.000000
C	-01.822629	+00.362901	+00.000000
C	+03.105828	+00.276382	+00.000000
C	-03.105828	-00.276382	+00.000000

C	+04.289540	-00.394078	+00.000000
C	-04.289540	+00.394078	+00.000000
C	+05.566133	+00.266047	+00.000000
C	-05.566133	-00.266047	+00.000000
C	+06.762788	-00.368733	+00.000000
C	-06.762788	+00.368733	+00.000000
H	+00.664910	+01.408177	+00.000000
H	-00.664910	-01.408177	+00.000000
H	+01.802750	-01.453948	+00.000000
H	-01.802750	+01.453948	+00.000000
H	+03.121762	+01.367930	+00.000000
H	-03.121762	-01.367930	+00.000000
H	+04.283850	-01.484557	+00.000000
H	-04.283850	+01.484557	+00.000000
H	+05.558560	+01.358466	+00.000000
H	-05.558560	-01.358466	+00.000000
H	+06.837173	-01.454218	+00.000000
H	-06.837173	+01.454218	+00.000000
C	+08.012597	+00.388036	+00.000000
H	+07.895355	+01.492923	+00.000000
C	-08.012597	-00.388036	+00.000000
H	-07.895355	-01.492923	+00.000000
O	-09.129372	+00.124944	+00.000000
O	+09.129372	-00.124944	+00.000000

XYZ Geometry CO_9

C	+00.591054	-00.339333	+00.000000
C	-00.591054	+00.339333	+00.000000

C	+01.873727	+00.296665	+00.000000
C	-01.873727	-00.296665	+00.000000
C	+03.057032	-00.378837	+00.000000
C	-03.057032	+00.378837	+00.000000
C	+04.337037	+00.265573	+00.000000
C	-04.337037	-00.265573	+00.000000
C	+05.524580	-00.398672	+00.000000
C	-05.524580	+00.398672	+00.000000
C	+06.797169	+00.268381	+00.000000
C	-06.797169	-00.268381	+00.000000
C	+07.997995	-00.358825	+00.000000
C	-07.997995	+00.358825	+00.000000
H	+00.570006	-01.430629	+00.000000
H	-00.570006	+01.430629	+00.000000
H	+01.893358	+01.388131	+00.000000
H	-01.893358	-01.388131	+00.000000
H	+03.041317	-01.469948	+00.000000
H	-03.041317	+01.469948	+00.000000
H	+04.347927	+01.357264	+00.000000
H	-04.347927	-01.357264	+00.000000
H	+05.524710	-01.489164	+00.000000
H	-05.524710	+01.489164	+00.000000
H	+06.783149	+01.360785	+00.000000
H	-06.783149	-01.360785	+00.000000
H	+08.079241	-01.443811	+00.000000
H	-08.079241	+01.443811	+00.000000
C	+09.242564	+00.405879	+00.000000

H	+09.117786	+01.510015	+00.000000
C	-09.242564	-00.405879	+00.000000
H	-09.117786	-01.510015	+00.000000
O	-10.363033	+00.099214	+00.000000
O	+10.363033	-00.099214	+00.000000

XYZ Geometry CO_{10}

C	+00.640886	+00.317778	+00.000000
C	-00.640886	-00.317778	+00.000000
C	+01.824716	-00.359050	+00.000000
C	-01.824716	+00.359050	+00.000000
C	+03.105535	+00.279438	+00.000000
C	-03.105535	-00.279438	+00.000000
C	+04.291285	-00.392386	+00.000000
C	-04.291285	+00.392386	+00.000000
C	+05.568782	+00.256255	+00.000000
C	-05.568782	-00.256255	+00.000000
C	+06.759220	-00.403131	+00.000000
C	-06.759220	+00.403131	+00.000000
C	+08.028750	+00.269245	+00.000000
C	-08.028750	-00.269245	+00.000000
C	+09.232666	-00.352241	+00.000000
C	-09.232666	+00.352241	+00.000000
H	+00.661357	+01.409222	+00.000000
H	-00.661357	-01.409222	+00.000000
H	+01.805569	-01.450374	+00.000000
H	-01.805569	+01.450374	+00.000000
H	+03.122429	+01.371031	+00.000000

H	-03.122429	-01.371031	+00.000000
H	+04.279073	-01.483539	+00.000000
H	-04.279073	+01.483539	+00.000000
H	+05.575626	+01.348031	+00.000000
H	-05.575626	-01.348031	+00.000000
H	+06.763863	-01.493611	+00.000000
H	-06.763863	+01.493611	+00.000000
H	+08.009827	+01.361610	+00.000000
H	-08.009827	-01.361610	+00.000000
H	+09.319039	-01.436827	+00.000000
H	-09.319039	+01.436827	+00.000000
C	+10.473323	+00.418343	+00.000000
H	+10.343002	+01.521877	+00.000000
C	-10.473323	-00.418343	+00.000000
H	-10.343002	-01.521877	+00.000000
O	-11.596470	+00.080935	+00.000000
O	+11.596470	-00.080935	+00.000000

XYZ Geometry CO_{11}

C	-00.000963	+00.682863	+00.000000
C	+01.187500	+01.481569	+00.000000
C	+00.000963	-00.682863	+00.000000
C	+01.187500	+02.847039	+00.000000
C	-01.187500	-01.481569	+00.000000
C	+02.379058	+03.642286	+00.000000
C	-01.187500	-02.847039	+00.000000
C	+02.385397	+05.006792	+00.000000
C	-02.379058	-03.642286	+00.000000

C	+03.584809	+05.793287	+00.000000
C	-02.385397	-05.006792	+00.000000
C	+03.605045	+07.155593	+00.000000
C	-03.584809	-05.793287	+00.000000
C	+04.821542	+07.922767	+00.000000
C	-03.605045	-07.155593	+00.000000
C	+04.881740	+09.277874	+00.000000
H	-00.959510	+01.205885	+00.000000
H	+02.145742	+00.957776	+00.000000
H	+00.959510	-01.205885	+00.000000
H	+00.230389	+03.372458	+00.000000
H	-02.145742	-00.957776	+00.000000
H	+03.335116	+03.114373	+00.000000
H	-00.230389	-03.372458	+00.000000
H	+01.432314	+05.538917	+00.000000
H	-03.335116	-03.114373	+00.000000
H	+04.535628	+05.255884	+00.000000
H	-01.432314	-05.538917	+00.000000
H	+02.661258	+07.702579	+00.000000
H	-04.535628	-05.255884	+00.000000
H	+05.759779	+07.362289	+00.000000
H	-02.661258	-07.702579	+00.000000
H	+03.983801	+09.892923	+00.000000
C	-04.821542	-07.922767	+00.000000
H	-05.759779	-07.362289	+00.000000
C	+06.168649	+09.971682	+00.000000
H	+07.061994	+09.310318	+00.000000

O	+06.293169	+11.195539	+00.000000
C	-04.881740	-09.277874	+00.000000
H	-03.983801	-09.892923	+00.000000
C	-06.168649	-09.971682	+00.000000
H	-07.061994	-09.310318	+00.000000
O	-06.293169	-11.195539	+00.000000

XYZ Geometry CO_{12}

C	+00.000764	+00.715758	+00.000000
C	-00.000764	-00.715758	+00.000000
C	+01.135725	+01.475989	+00.000000
C	-01.135725	-01.475989	+00.000000
C	+01.135725	+02.907653	+00.000000
C	-01.135725	-02.907653	+00.000000
C	+02.268679	+03.670103	+00.000000
C	-02.268679	-03.670103	+00.000000
C	+02.263060	+05.102490	+00.000000
C	-02.263060	-05.102490	+00.000000
C	+03.391223	+05.870206	+00.000000
C	-03.391223	-05.870206	+00.000000
C	+03.373237	+07.304275	+00.000000
C	-03.373237	-07.304275	+00.000000
C	+04.491480	+08.082711	+00.000000
C	-04.491480	-08.082711	+00.000000
H	-00.967636	+01.220352	+00.000000
H	+00.967636	-01.220352	+00.000000
H	+02.104381	+00.971994	+00.000000
H	-02.104381	-00.971994	+00.000000

H	+00.166351	+03.410635	+00.000000
H	-00.166351	-03.410635	+00.000000
H	+03.238939	+03.169384	+00.000000
H	-03.238939	-03.169384	+00.000000
H	+01.291142	+05.600673	+00.000000
H	-01.291142	-05.600673	+00.000000
H	+04.365066	+05.377110	+00.000000
H	-04.365066	-05.377110	+00.000000
H	+02.396154	+07.792344	+00.000000
H	-02.396154	-07.792344	+00.000000
H	+05.472641	+07.606013	+00.000000
H	-05.472641	-07.606013	+00.000000
C	+04.447391	+09.520158	+00.000000
H	+03.458053	+09.984554	+00.000000
C	-04.447391	-09.520158	+00.000000
H	-03.458053	-09.984554	+00.000000
C	+05.537067	+10.328025	+00.000000
H	+06.549140	+09.927679	+00.000000
C	+05.392376	+11.782762	+00.000000
H	+04.344397	+12.153246	+00.000000
O	+06.337080	+12.570758	+00.000000
C	-05.537067	-10.328025	+00.000000
H	-06.549140	-09.927679	+00.000000
C	-05.392376	-11.782762	+00.000000
H	-04.344397	-12.153246	+00.000000
O	-06.337080	-12.570758	+00.000000

XYZ Geometry CO_{13}

C	-00.000585	+00.683196	+00.000000
C	+01.186839	+01.481923	+00.000000
C	+00.000585	-00.683196	+00.000000
C	+01.186839	+02.848174	+00.000000
C	-01.186839	-01.481923	+00.000000
C	+02.376205	+03.644642	+00.000000
C	-01.186839	-02.848174	+00.000000
C	+02.380212	+05.010392	+00.000000
C	-02.376205	-03.644642	+00.000000
C	+03.574052	+05.801705	+00.000000
C	-02.380212	-05.010392	+00.000000
C	+03.585849	+07.166339	+00.000000
C	-03.574052	-05.801705	+00.000000
C	+04.788340	+07.947770	+00.000000
C	-03.585849	-07.166339	+00.000000
C	+04.815026	+09.310065	+00.000000
H	-00.959361	+01.205993	+00.000000
H	+02.145422	+00.958660	+00.000000
H	+00.959361	-01.205993	+00.000000
H	+00.228941	+03.372405	+00.000000
H	-02.145422	-00.958660	+00.000000
H	+03.333380	+03.118712	+00.000000
H	-00.228941	-03.372405	+00.000000
H	+01.424661	+05.538618	+00.000000
H	-03.333380	-03.118712	+00.000000
H	+04.528289	+05.270399	+00.000000
H	-01.424661	-05.538618	+00.000000

H	+02.634907	+07.702270	+00.000000
H	-04.528289	-05.270399	+00.000000
H	+05.736817	+07.406167	+00.000000
H	-02.634907	-07.702270	+00.000000
H	+03.873842	+09.861510	+00.000000
C	-04.788340	-07.947770	+00.000000
H	-05.736817	-07.406167	+00.000000
C	+06.035081	+10.071292	+00.000000
H	+06.970501	+09.506074	+00.000000
C	-04.815026	-09.310065	+00.000000
H	-03.873842	-09.861510	+00.000000
C	-06.035081	-10.071292	+00.000000
H	-06.970501	-09.506074	+00.000000
C	+06.102444	+11.426134	+00.000000
H	+05.207755	+12.045888	+00.000000
C	+07.392866	+12.112989	+00.000000
H	+08.282579	+11.446679	+00.000000
O	+07.524358	+13.336171	+00.000000
C	-06.102444	-11.426134	+00.000000
H	-05.207755	-12.045888	+00.000000
C	-07.392866	-12.112989	+00.000000
H	-08.282579	-11.446679	+00.000000
O	-07.524358	-13.336171	+00.000000

S3 Additional tables and figures

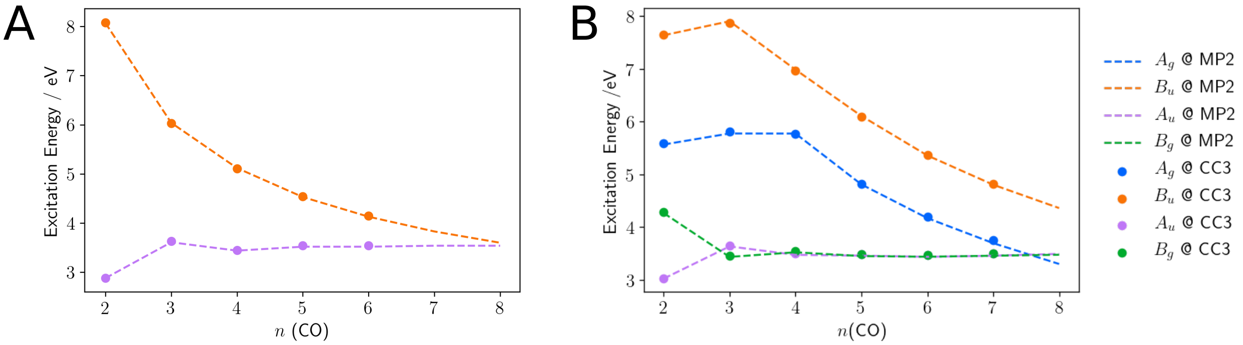

Figure S1: Excitation energies of CO_n systems computed at CC3 and CAS-SCF level (panel A and B respectively) on CC3 structures (dots) and MP2 structures (dashed lines).

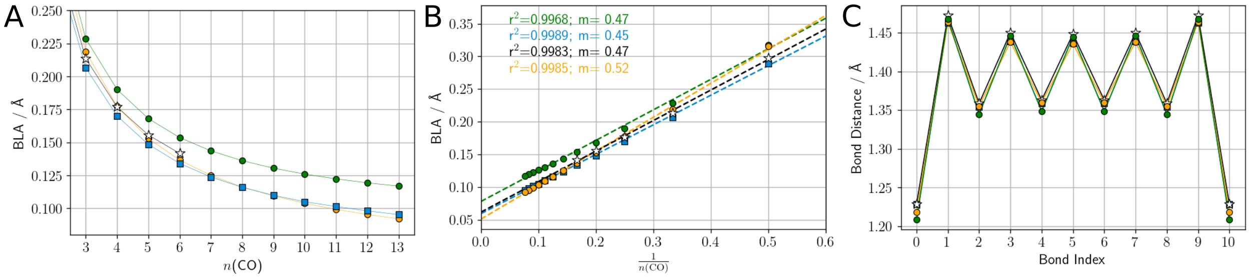

Figure S2: (A) Trends in BLA against length of the CO_n systems computed at different levels of theory: CC3 (stars), MP2 (blue squares), and DFT with the B3LYP and CAM-B3LYP functionals (orange and green circles respectively). (B) Linear regression of BLA data computed with different methods *versus* $\frac{1}{n(\mathrm{CO})}$ showing a clear inverse proportionality between the BLA and n; r^2 and slope (m) are reported for each method. (C) Individual bond lengths of CO_6 computed at the optimized geometry with different methods.

Table S3: Analysis of the converged DMRG wavefunctions for the low-lying electronic states of CO_{13} in terms of dominant Slater determinant contributions. For each state the determinants having coefficients with absolute value larger than 0.1 are listed. The determinants strings uses the following code: '2' doubly occupied, 'u' one electron spin up, 'd' one electron spin down, '0' empty. Each Slater determinant is either classified as Hartree-Fock (HF), singly excited (sing.) or doubly excited (doub.).

State	Determinant	Coefficient	Det. Type
GS	2222222222222220000000000000	-0.77	HF
A_g	2222222222222202000000000000	-0.49	doub.
	2222222222222u2d000000000000	-0.17	sing.
	2222222222222d2u000000000000	+0.17	sing.
	22222222222222d0u00000000000	+0.16	sing.
	22222222222222u0d00000000000	-0.16	sing.
	222222222222220d0u0000000000	-0.14	doub.
	222222222222220u0d0000000000	+0.14	doub.
	2222222222222uddu00000000000	+0.12	doub.
	2222222222222duud00000000000	+0.12	doub.
	2222222222222022000000000000	+0.11	doub.
	2222222222222dduu00000000000	-0.11	doub.
	2222222222222uudd00000000000	-0.11	doub.
A_u	222222222d222220u00000000000	-0.30	sing.
	222222222u222220d00000000000	+0.30	sing.
	22222222d22222200u0000000000	+0.29	sing.
	22222222u22222200d0000000000	-0.29	sing.
	22222222d222222u000000000000	+0.23	sing.
	22222222u222222d000000000000	-0.23	sing.
	222222222d222220000000u00000	-0.16	sing.

(Continued on next page)

State	Determinant	Coefficient	Det. Type
A_u	222222222u222220000000d00000	+0.16	sing.
	222222222u22222000000d000000	-0.15	sing.
	222222222d22222000000u000000	+0.15	sing.
	22222222d2222220000000000u0	-0.11	sing.
	22222222u2222220000000000d0	+0.11	sing.
B_g	22222222d2222220u00000000000	+0.30	sing.
	22222222u2222220d00000000000	-0.30	sing.
	222222222u2222200d0000000000	+0.29	sing.
	222222222d2222200u0000000000	-0.29	sing.
	222222222d22222u000000000000	-0.23	sing.
	222222222u22222d000000000000	+0.23	sing.
	22222222u2222220000000d00000	-0.16	sing.
	22222222d2222220000000u00000	+0.16	sing.
	22222222u222222000000d000000	+0.15	sing.
	22222222d222222000000u000000	-0.15	sing.
	222222222u2222200000000000d0	-0.11	sing.
	222222222d2222200000000000u0	+0.11	sing.
B_u^-	2222222222222du2000000000000	+0.27	doub.
	2222222222222ud2000000000000	-0.27	doub.
	22222222222220du00000000000	+0.22	doub.
	22222222222220ud00000000000	-0.22	doub.
	222222222222u22d000000000000	+0.22	sing.
	222222222222d22u000000000000	-0.22	sing.
	2222222222222d00u0000000000	+0.16	sing.

(Continued on next page)

State	Determinant	Coefficient	Det. Type
B_u^-	22222222222222u00d0000000000	-0.16	sing.
B_u^+	22222222222222du000000000000	+0.56	sing.
	22222222222222ud000000000000	-0.56	sing.
	2222222222222d20u00000000000	-0.12	sing.
	2222222222222u20d00000000000	+0.12	sing.

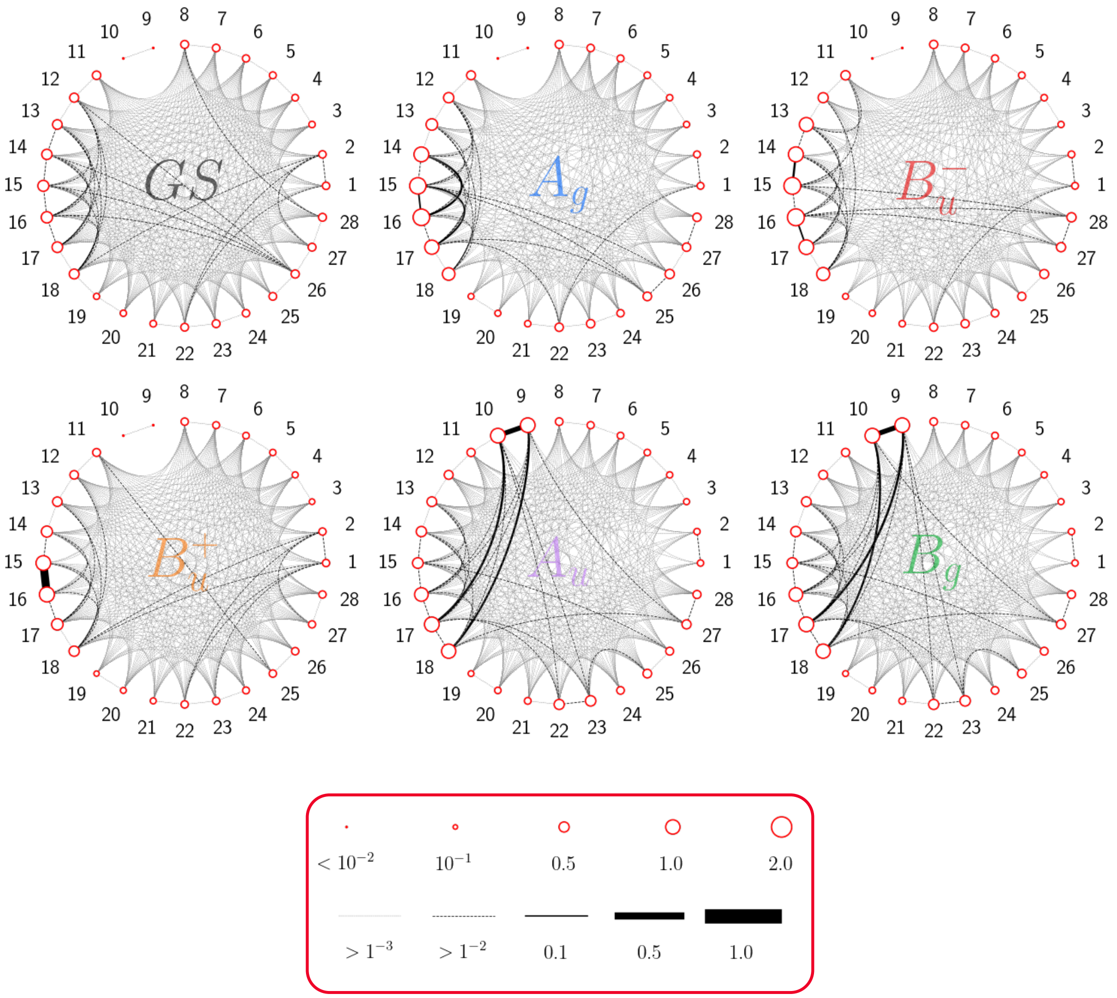

Figure S3: Mutual Information (MI) diagrams of CO_{13} computed from the DMRG-SCF converged wavefunctions; each dot corresponds to an active space orbital (the index number is the same as used in Figure S4) and its size is proportional to its single orbital entropy; the style and shape of the line connecting two dots is proportional to the mutual information of the two.

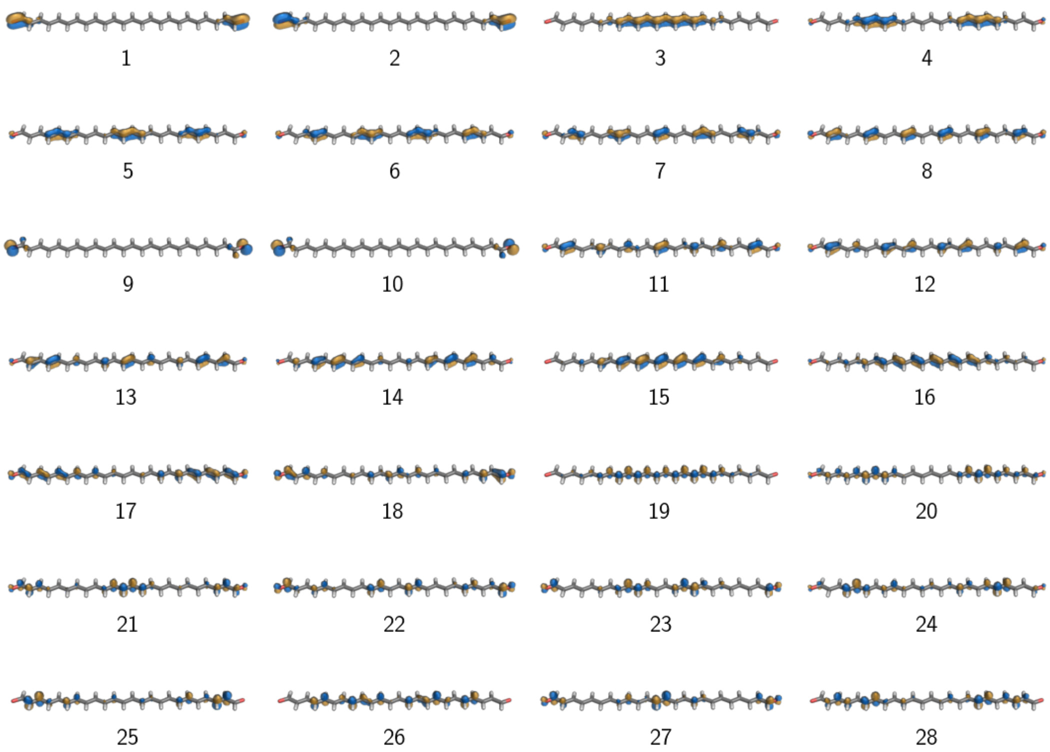

Figure S4: Optimized active space orbitals for CO_{13}; orbitals are represented as isosurfaces at two arbitrary values (+0.001 yellow, -0.001 blue). Orbitals 9 and 10 are the n orbitals with B_u and A_g symmetries respectively; all the other orbitals are π orbitals with A_u and B_g symmetries.

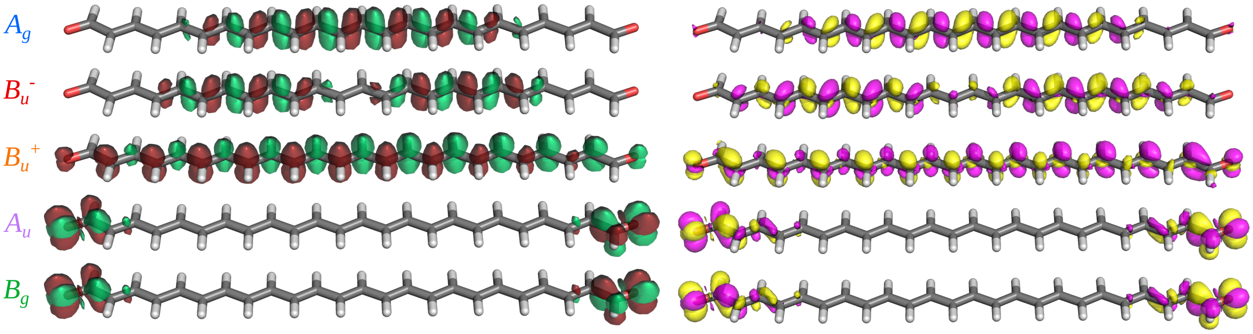

Figure S5: Ground-to-excited state transition densities of CO_{13} computed at DMRG-SCF (left) and TD-B3LYP level (right). From top to bottom: A_g, B_u^-, B_u^+, A_u and B_g. Transition densities are represented as isodensity surfaces at ±0.001.

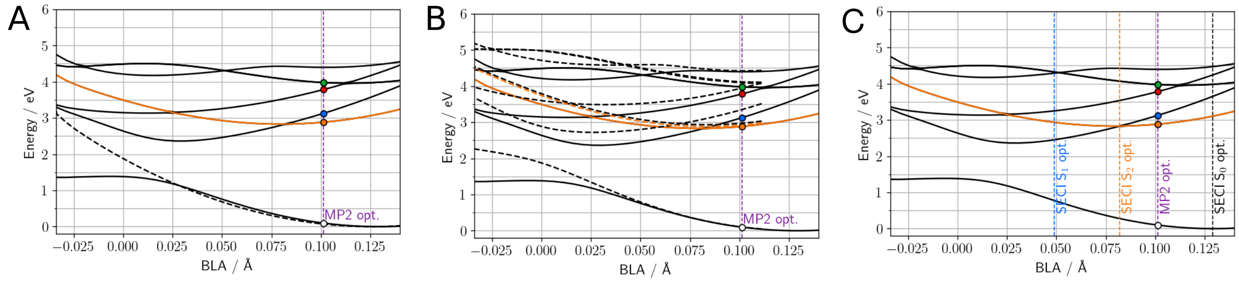

Figure S6: Evolution of the energies of CO_{11} along the BLA coordinate. In all plots the solid lines refer to SECI energies calculated at MP2 geometries and they are the same reported in the main text while in (A) the dashed line refers to GS energies computed at OM2/HF level on the MP2 geometries; in (B) the dashed lines refer to SECI energies calculated at SECI geometries; in (c) the dashed vertical lines indicate the BLA values of the SECI optimized geometry for S_0, S_1 and S_2.

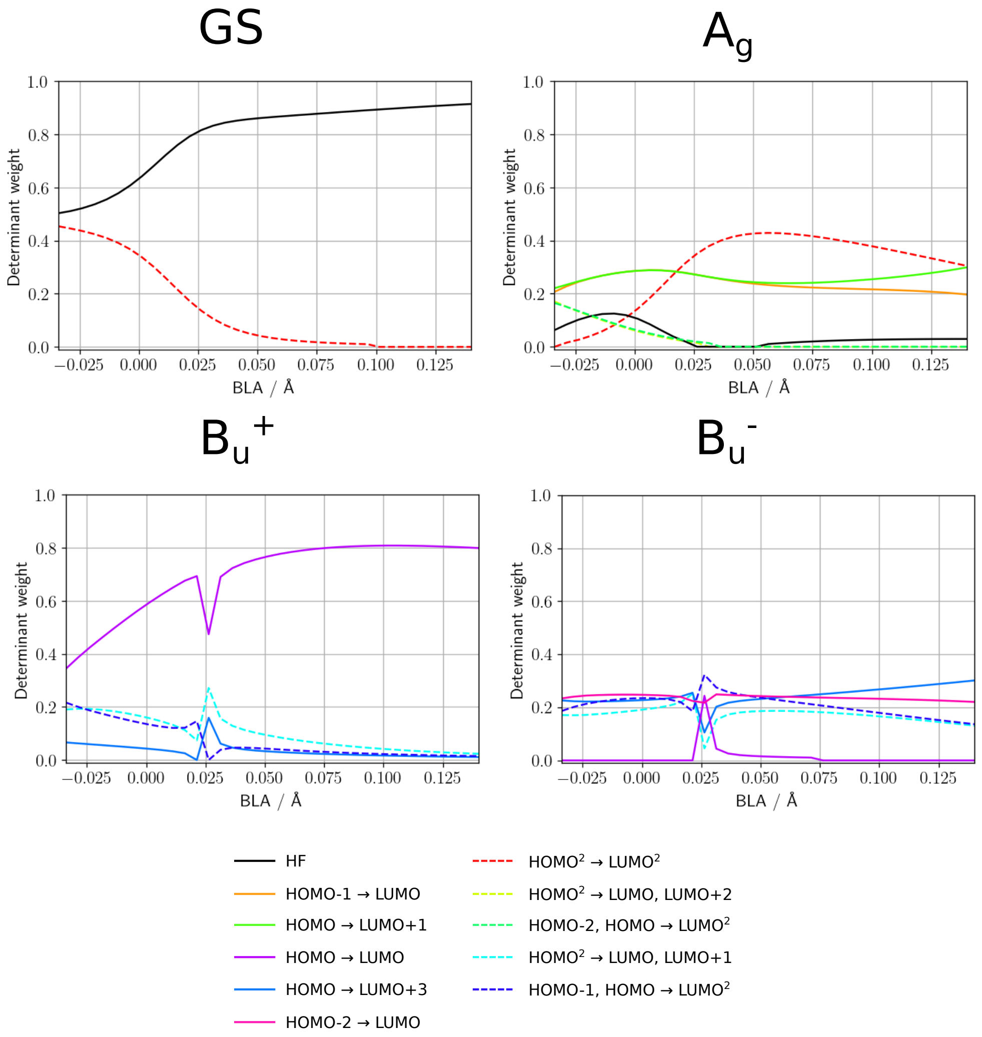

Figure S7: Evolution of main determinant contributions to the SECI wavefunction along the BLA coordinate for the three lowest lying electronic states. Each determinant is represented with a different color; solid lines are used for HF and singly excited determinants while dashed lines are used for doubly excited determinants. The discontinuities can be due to the orbital switching/mixing during the geometry change.

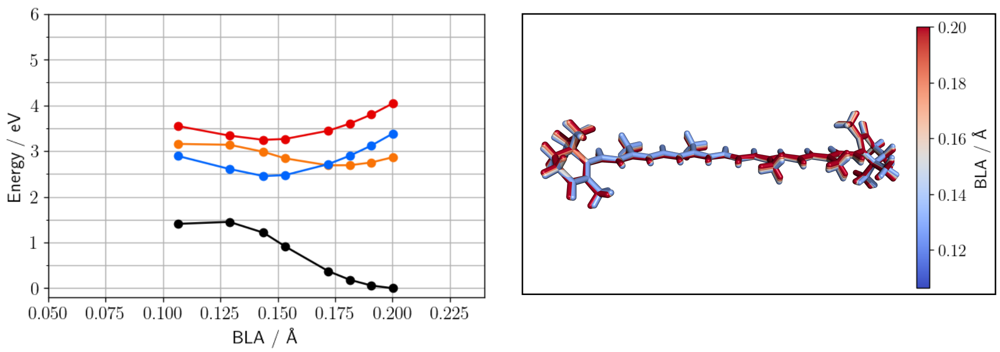

Figure S8: Ground and $\pi \to \pi^*$ excited states of CAN computed at SECI level (left panel) on BLA scan performed at MP2 level in vacuum (right).

References

(S1) Christiansen, O.; Koch, H.; Jørgensen, P. Response Functions in the CC3 Iterative Triple Excitation Model. *J. Chem. Phys.* **1995**, *103*, 7429–7441.

(S2) Koch, H.; Christiansen, O.; Jorgensen, P.; Sanchez de Merás, A. M.; Helgaker, T. The CC3 Model: An Iterative Coupled Cluster Approach Including Connected Triples. *J. Chem. Phys.* **1997**, *106*, 1808–1818.

(S3) Matthews, D. A.; Cheng, L.; Harding, M. E.; Lipparini, F.; Stopkowicz, S.; Jagau, T.-C.; Szalay, P. G.; Gauss, J.; Stanton, J. F. Coupled-Cluster Techniques for Computational Chemistry: The CFOUR Program Package. *J. Chem. Phys.* **2020**, *152*, 214108.

(S4) Véril, M.; Scemama, A.; Caffarel, M.; Lipparini, F.; Boggio-Pasqua, M.; Jacquemin, D.; Loos, P.-F. QUESTDB: a Database of Highly-Accurate Excitation Energies for the Electronic Structure Community. *WIREs Comput. Mol. Sci.* **2021**, *11*, e1517.

(S5) Loos, P.-F.; Boggio-Pasqua, M.; Scemama, A.; Caffarel, M.; Jacquemin, D. Reference Energies for Double Excitations. *J. Chem. Theory Comput.* **2019**, *15*, 1939–1956.

(S6) Sun, Q.; Berkelbach, T. C.; Blunt, N. S.; Booth, G. H.; Guo, S.; Li, Z.; Liu, J.; McClain, J. D.; Sayfutyarova, E. R.; Sharma, S.; Wouters, S.; Chan, G. K.-L. PySCF: the Python-based Simulations of Chemistry Framework. *WIREs Comput. Mol. Sci.* **2018**, *8*, e1340.

(S7) Sun, Q. Libcint: An Efficient General Integral Library for Gaussian Basis Functions. *J. Comput. Chem.* **2015**, *36*, 1664–1671.

(S8) Sun, Q.; Yang, J.; Chan, G. K.-L. A General Second Order Complete Active Space Self-Consistent-Field Solver for Large-Scale Systems. *Chem. Phys. Lett.* **2017**, *683*, 291 – 299.

(S9) Olivares-Amaya, R.; Hu, W.; Nakatani, N.; Sharma, S.; Yang, J.; Chan, G. K.-L. The ab-initio Density Matrix Renormalization Group in Practice. *J. Chem. Phys.* **2015**, *142*, 034102.

(S10) Knizia, G. Intrinsic Atomic Orbitals: An Unbiased Bridge between Quantum Theory and Chemical Concepts. *J. Chem. Theory Comput.* **2013**, *9*, 4834–4843.

(S11) Derricotte, W. D.; Evangelista, F. A. Localized Intrinsic Valence Virtual Orbitals as a Tool for the Automatic Classification of Core Excited States. *J. Chem. Theory Comput.* **2017**, *13*, 5984–5999.

(S12) Subotnik, J. E.; Dutoi, A. D.; Head-Gordon, M. Fast Localized Orthonormal Virtual Orbitals which Depend Smoothly on Nuclear Coordinates. *J. Chem. Phys.* **2005**, *123*, 114108.

(S13) Keal, T. W.; Wanko, M.; Thiel, W. Assessment of Semiempirical Methods for the Photoisomerisation of a Protonated Schiff Base. *Theor. Chem. Acc.* **2009**, *123*, 145–156.

(S14) Ivanic, J.; Ruedenberg, K. Rotation Matrices for Real Spherical Harmonics. Direct Determination by Recursion. *J. Phys. Chem.* **1996**, *100*, 6342–6347.

(S15) Frisch, M. J.; Trucks, G. W.; Schlegel, H. B.; Scuseria, G. E.; Robb, M. A.; Cheeseman, J. R.; Scalmani, G.; Barone, V.; Petersson, G. A.; Nakatsuji, H.; Li, X.; Caricato, M.; Marenich, A. V.; Bloino, J.; Janesko, B. G.; Gomperts, R.; Mennucci, B.; Hratchian, H. P.; Ortiz, J. V.; Izmaylov, A. F.; Sonnenberg, J. L.; Williams-Young, D.; Ding, F.; Lipparini, F.; Egidi, F.; Goings, J.; Peng, B.; Petrone, A.; Henderson, T.; Ranasinghe, D.; Zakrzewski, V. G.; Gao, J.; Rega, N.; Zheng, G.; Liang, W.; Hada, M.; Ehara, M.; Toyota, K.; Fukuda, R.; Hasegawa, J.; Ishida, M.; Nakajima, T.; Honda, Y.; Kitao, O.; Nakai, H.; Vreven, T.; Throssell, K.; Montgomery, J. A., Jr.; Peralta, J. E.; Ogliaro, F.; Bearpark, M. J.; Heyd, J. J.; Brothers, E. N.; Kudin, K. N.; Staroverov, V. N.; Keith, T. A.; Kobayashi, R.; Normand, J.; Raghavachari, K.; Rendell, A. P.; Burant, J. C.; Iyengar, S. S.; Tomasi, J.; Cossi, M.; Millam, J. M.; Klene, M.; Adamo, C.; Cammi, R.; Ochterski, J. W.; Martin, R. L.; Morokuma, K.; Farkas, O.; Foresman, J. B.; Fox, D. J. Gaussian˜16 Revision A.03. 2016; Gaussian Inc. Wallingford CT.

(S16) Becke, A. D. Density-functional thermochemistry. III. The role of exact exchange. *J. Chem. Phys.* **1993**, *98*, 5648–6.

(S17) Stephens, P. J.; Devlin, F. J.; Chabalowski, C. F.; Frisch, M. J. Ab Initio Calculation of Vibrational Absorption and Circular Dichroism Spectra Using Density Functional Force Fields. *J. Phys. Chem.* **1994**, *98*, 11623–11627.

(S18) Yanai, T.; Tew, D. P.; Handy, N. C. A new Hybrid Exchange–Correlation Functional using the Coulomb-Attenuating Method (CAM-B3LYP). *Chem. Phys. Lett.* **2004**, *393*, 51–57.

(S19) Chung, L. W.; Sameera, W. M. C.; Ramozzi, R.; Page, A. J.; Hatanaka, M.;

Petrova, G. P.; Harris, T. V.; Li, X.; Ke, Z.; Liu, F.; Li, H.-B.; Ding, L.; Morokuma, K. The ONIOM Method and Its Applications. *Chem. Rev.* **2015**, *115*, 5678–5796.

(S20) Bondanza, M.; Cupellini, L.; Lipparini, F.; Mennucci, B. The Multiple Roles of the Protein in the Photoactivation of Orange Carotenoid Protein. *Chem* **2020**, *6*, 187–203.
